# ENABLE-SG (Educate, Nurture, Advise, Before Life Ends for Singapore) as a proactive palliative care model: protocol for a hybrid type 1 effectiveness-implementation randomized wait-list controlled trial

**DOI:** 10.1186/s12904-024-01353-2

**Published:** 2024-01-30

**Authors:** Yu Ke, Yin Bun Cheung, Marie Bakitas, J. Nicholas Odom, Elaine Lum, Daniel Shao Weng Tan, Tira J. Tan, Eric Finkelstein, Hong Choon Oh, Siqin Zhou, Grace Meijuan Yang

**Affiliations:** 1 Division of Supportive and Palliative Care, National Cancer Centre Singapore, 30 Hospital Boulevard, Singapore, 168583 Singapore; 2 Centre for Quantitative Medicine, Duke-NUS Medical School, Singapore, Singapore; 3 Tampere Center for Child, Adolescent and Maternal Health Research, Tampere University, Tampere, Finland; 4 Duke-NUS Medical School, Program in Health Services & Systems Research, Singapore, Singapore; 5 School of Nursing, University of Alabama at Birmingham (UAB), Birmingham, AL USA; 6 Division of Geriatrics, Gerontology, and Palliative Care, Department of Medicine, UAB Centre for Palliative and Supportive Care, Birmingham, AL USA; 7 School of Clinical Sciences, Queensland University of Technology, Brisbane, QLD Australia; 8 Centre for Population Health Research & Implementation, SingHealth, Singapore, Singapore; 9 Division of Medical Oncology, National Cancer Centre Singapore, Singapore, Singapore; 10 Cancer Therapeutics Research Laboratory, National Cancer Centre Singapore, Singapore, Singapore; 11 Genome Institute of Singapore, A*Star, Singapore, Singapore; 12 Duke-NUS Medical School, Oncology Academic Clinical Program, Singapore, Singapore; 13 Duke-NUS Medical School, Lien Centre for Palliative Care, Singapore, Singapore; 14 Health Services Research, Changi General Hospital, Singapore, Singapore; 15 Division of Clinical Trials and Epidemiological Sciences, National Cancer Centre, Singapore, Singapore

**Keywords:** Cancer, Palliative care, Psychoeducational, Telehealth, Health coaching, Implementation

## Abstract

**Background:**

Specialist palliative care is often provided late in the patient’s disease trajectory in response to uncontrolled symptoms. Shifting from this reactionary illness-stress paradigm to a proactive health-wellness approach, the ENABLE (Educate, Nurture, Advise, Before Life Ends) telehealth model aims to enhance the coping, stress and symptom management, self-care, and advance care planning skills of patients with advanced cancers and their caregivers. The ENABLE model has been culturally adapted to Singapore (ENABLE-SG) and pilot-tested. A hybrid type 1 effectiveness-implementation design will be used to evaluate the effectiveness of ENABLE-SG while collecting real-world implementation data.

**Methods:**

This single-centre, assessor-blind, wait-list (immediately vs. 6 months) randomized controlled trial will recruit 300 adult patients within 60 days of an advanced cancer diagnosis and their family caregivers from the National Cancer Centre of Singapore. ENABLE-SG comprises structured psychoeducational sessions with a telehealth coach, covering essential topics of early palliative care. Participants will be assessed at baseline and every 3 months until patient’s death, 12 months (caregivers), or end of study (patients). The primary outcome is patient quality of life 6 months after baseline. Secondary patient-reported outcomes include mood, coping, palliative care concerns, and health status. Secondary caregiver-reported outcomes include caregiver quality of life, mood, coping, and care satisfaction. Mixed-effects regression modelling for repeated measurements will be used. To assess the effectiveness of ENABLE-SG versus usual care, patient and caregiver outcomes at 6 months will be compared. To compare earlier versus delayed ENABLE-SG, patient and caregiver outcomes at 12 months will be compared. Within the hybrid type 1 effectiveness-implementation design, implementation outcomes will be evaluated in both the early and delayed groups. Acceptability, adoption, appropriateness, and feasibility will be assessed using a feedback survey and semi-structured interviews with a purposive sample of patients, caregivers, and healthcare providers. Transcribed interviews will be analysed thematically. Other implementation outcomes of penetration, fidelity, and cost will be assessed using records of study-related processes and summarized using descriptive statistics. A cost-effectiveness analysis will also be conducted.

**Discussion:**

This study will assess both effectiveness and implementation of ENABLE-SG. Insights into implementation processes can facilitate model expansion and upscaling.

**Trial registration:**

Registered prospectively on ClinicalTrials.gov, NCT06044441. Registered on 21/09/2023.

**Supplementary Information:**

The online version contains supplementary material available at 10.1186/s12904-024-01353-2.

## Background

Worldwide and in Singapore, cancer is a prevalent cause of death [1, 2]. With Singapore’s ageing population and a higher risk of cancer associated with increasing age, the number of people diagnosed and living with cancer is projected to rise [3]. Palliative care addresses the burden of advanced cancer experienced by patients and their families through early identification, assessment, and treatment of physical, psychosocial, and spiritual sources of poor quality of life [4–7]. Both the American Society for Clinical Oncology and the European Society for Medical Oncology recommend that all patients with advanced cancer receive palliative care from the time of diagnosis [8, 9].

Despite guideline recommendations, the current practice of specialist palliative care in Singapore focuses on supporting patients with complex problems in the last weeks of life [10]. Palliative care is often triggered by uncontrolled symptoms during crises, representing a reactive care approach [11, 12]. This approach delays palliative care initiation among patients in their early months of diagnosis when they may still have a reasonably good quality of life without overt symptoms or problems [13–16]. Models of palliative care should shift from the current reactionary illness-stress paradigm to a proactive health-wellness approach that is integrated early in the patient’s serious illness trajectory [17].

The hallmarks of early palliative are symptom management, coping skills development, treatment decision-making, and engagement in advance care planning [18]. Existing models often rely on specialist clinicians in outpatient settings to provide such comprehensive care, posing challenges to scalability and sustainability [19–24]. The ENABLE (Educate, Nurture, Advise, Before Life Ends) telehealth model developed in the United States (U.S.) offers a possibility of engaging non-specialists as health coaches to deliver early palliative care proactively [25–27]. Through structured telephonic sessions, health coaches can coach patients and their caregivers on coping, stress and symptom management, self-care, and advanced care planning skills, empowering them to mitigate and avoid crises. In the U.S., randomized controlled trials showed that compared to usual care, the ENABLE model improved the quality of life of patients with advanced cancer [28]. Patients also experienced additional survival benefits when enrolled early in the ENABLE model, with less depression and lower stress burden among caregivers [29, 30].

Although the ENABLE model has demonstrated effectiveness in the U.S., it is uncertain if the benefits will be transferrable cross-culturally when implemented in Singapore with different organisational, social, and cultural norms [31]. Addressing contextual differences, the ENABLE model has been culturally adapted (ENABLE-SG) through a qualitative formative evaluation. The main modifications made were adding screening questions for concerns salient to the local context and adjusting the content of coaching sessions to be relevant to the local population [32, 33]. The adapted ENABLE-SG was subsequently piloted in 43 patients and 15 caregivers (manuscript in preparation). The pilot study had a completion rate of 72% among patients and 94% among caregivers. Also, the telehealth mode of delivery was found acceptable, with 96% of the sessions conducted over the telephone. These findings demonstrate that ENABLE-SG is feasible and acceptable for further evaluation in the Singapore context.

Using a hybrid type 1 effectiveness-implementation design, this randomized wait-list controlled trial of the culturally adapted ENABLE-SG among patients with recently diagnosed advanced cancer and their caregivers will concurrently assess both effectiveness and implementation outcomes [34]. The simultaneous evaluation of clinical outcomes and implementation processes will facilitate a rapid translation of the model into clinical implementation subsequently to improve healthcare delivery. The specific aims are:

1. Assess the effectiveness of ENABLE-SG among patients with advanced cancer. At 6 months post-enrolment, we hypothesize that, compared to usual care (wait-list controls), patients who received ENABLE-SG will have better health-related quality of life (QoL) [primary outcome], mood, health status, coping strategies, fewer palliative care concerns, less acute healthcare utilisation, and smaller hospital bill [secondary outcomes]. At 12 months post-enrolment, we hypothesize that, compared to those who received delayed ENABLE-SG, patients who received early ENABLE-SG will report better abovementioned outcomes.
2. Assess the effectiveness of ENABLE-SG among caregivers of patients with advanced cancer. At 6 months post-enrolment, we hypothesize that, compared to usual care (wait-list controls), caregivers who received ENABLE-SG will have better health-related QoL, mood, coping strategies, satisfaction with care, and lower caregiving costs. At 12 months post-enrolment, we hypothesize that, compared to those who received delayed ENABLE-SG, caregivers who received early ENABLE-SG will report better abovementioned outcomes.
3. Assess ways to improve ENABLE-SG implementation in the real-world context by assessing its penetration, acceptability, adoption, appropriateness, feasibility, fidelity, and implementation cost.

## Methods/ design

### Study design

**Fig. 1:**
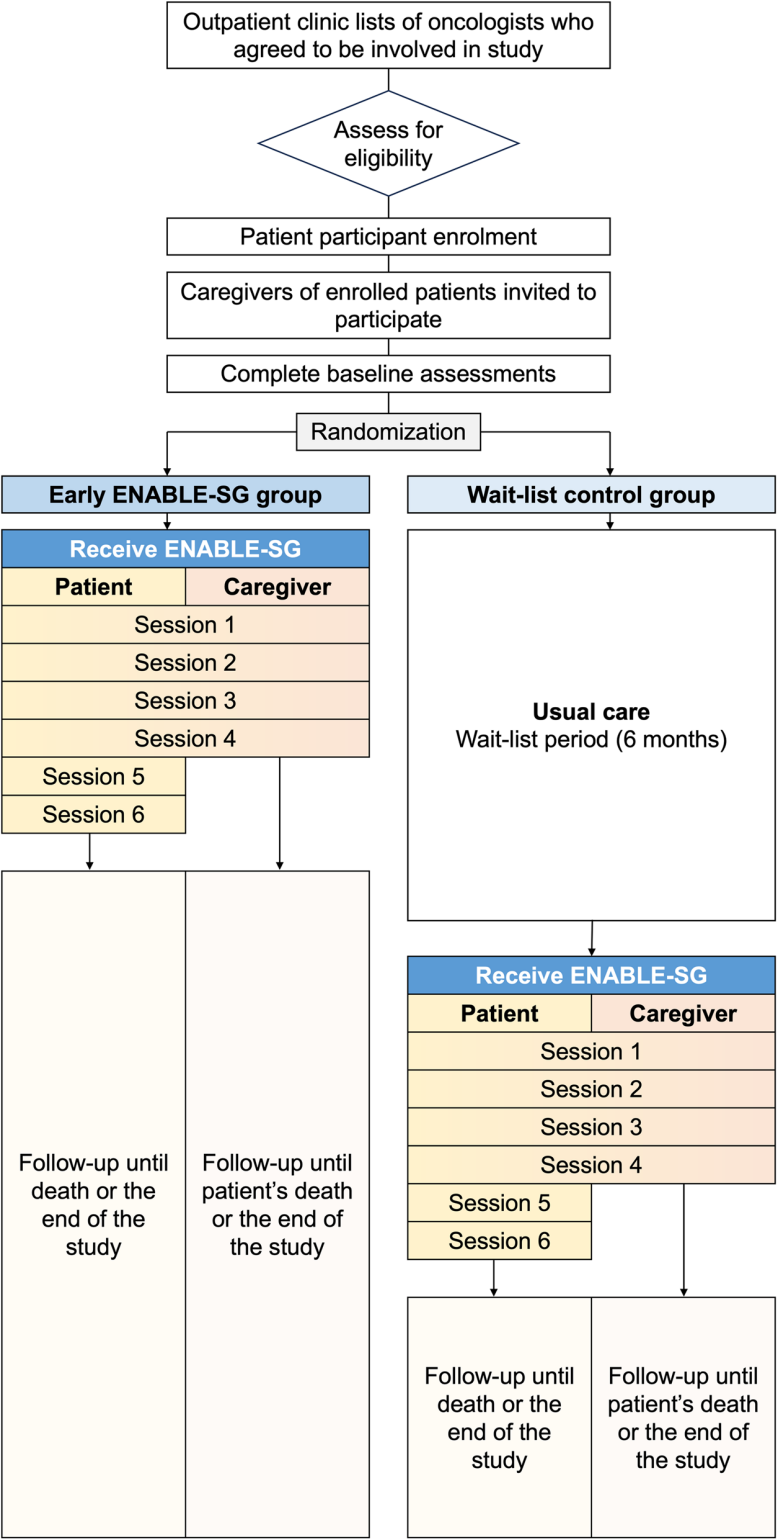
Study flow chart for trial participants Legend: Participants are randomized to either receive the ENABLE-SG intervention immediately (early ENABLE-SG group) or after 6 months (wait-list control group)

**Fig. 2:**
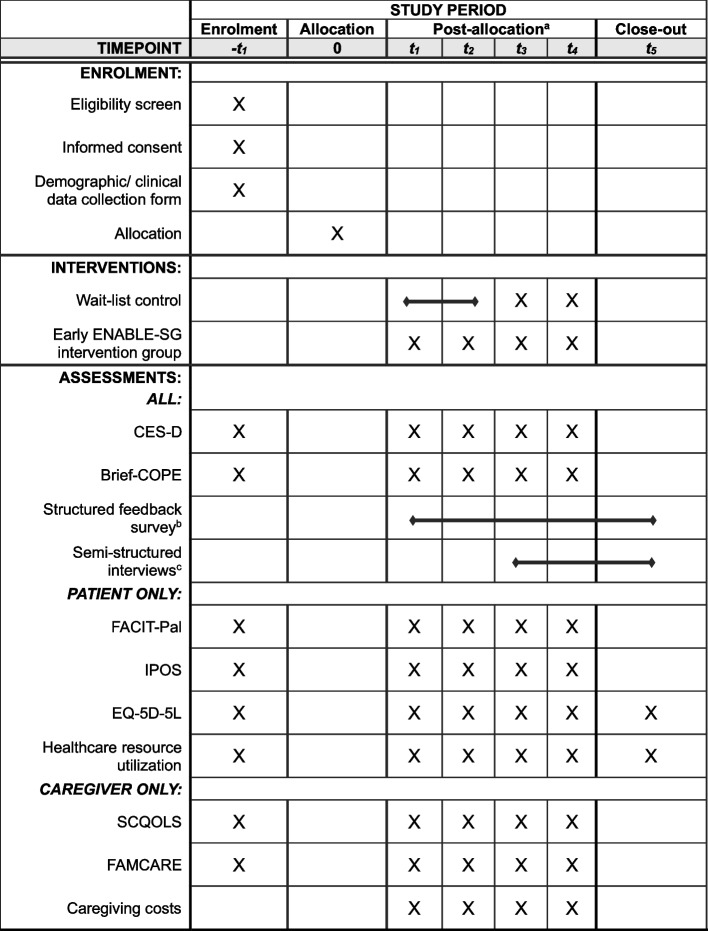
Overall study schedule for trial participants

This type 1 hybrid effectiveness-implementation randomized wait-list controlled trial will take place in the outpatient setting at the National Cancer Centre of Singapore (NCCS). NCCS serves 65% of all cancer patients in the public sector in Singapore. Patient participants will be randomized to receive the ENABLE-SG intervention immediately (early ENABLE-SG group) or after 6 months (wait-list control group) (Fig. 1). Caregiver participants will be assigned to the same group as the patient care recipient. Patient participants will be followed up until death or the end of the study, whichever is earlier. Caregiver participants will be followed up until the patient’s death or the end of study, whichever is earlier. Figure 2 depicts the study schedule.

### Participants

A patient will be eligible if he/she is (1) aged ≥ 21 years, (2) within 60 days of being informed of an advanced cancer diagnosis, defined as metastatic or recurrent/ progressive Stage III/IV solid tumour, (3) able to speak English or Mandarin Chinese, and (4) able to provide informed consent. Patients will be excluded if they (1) have a medical record documentation of an active severe mental illness, dementia, active suicidal ideation, uncorrected hearing loss, (2) are unable to complete patient-reported outcome measures, or (3) already known to a hospice or palliative care service. Patients without a caregiver or whose caregivers choose not to enrol may still participate in the study.

A caregiver will be eligible if he/she is (1) aged ≥ 21 years, (2) self-endorsing or identified by the enrolled patient with advanced cancer as an unpaid spouse/partner, relative or friend who knows the patient well, who provides regular support due to their cancer and who does not have to live in the same dwelling, (3) able to speak English or Mandarin Chinese, and (4) able to provide informed consent. Caregivers will be excluded if they are unable to complete caregiver-reported outcomes or have self-reported severe mental illness, dementia, active suicidal ideation, or uncorrected hearing loss.

To assess ways to improve ENABLE-SG implementation, healthcare providers whose patients are enrolled in the study will be invited to participate in semi-structured interviews. They will be eligible if they are aged ≥ 21 years and able to provide informed consent.

### Recruitment

The study team will screen NCCS outpatient clinic lists of participating oncologists for eligible patient participants. Potential patient participants will be approached in the oncology outpatient clinics to verify eligibility and obtain informed consent. Each enrolled patient participant will be asked to identify an eligible caregiver. A study team member will perform either in-person or remote consent-taking with eligible caregivers. Regardless of caregiver participation, the patient can still enrol in the study. Recruitment has started in December 2023.

### Randomization

After consent is obtained, patient participants will be randomized in a 1:1 ratio to the early ENABLE-SG intervention group or the wait-list control group using randomized permuted blocks generated by an independent statistician. The block size is concealed from the study team until study completion. Randomization will be stratified by tumour types (lung, gastrointestinal, breast, and others) to ensure comparable numbers allocated to both study groups. The consent taker will inform the randomizer after obtaining informed consent and administering baseline questionnaires. The randomizer will then notify the consent taker about the allocation status. The randomizer will not participate in any recruitment procedures.

### Usual care prior to delayed intervention

Patients randomized to the wait-list control group will receive usual care for the 6 months before receiving the ENABLE SG intervention. Under usual care, patients are managed by their primary oncologists who focus on cancer-directed treatments. When patients develop complex care needs, the primary oncologist may refer the patient to available palliative care services. Patients and their caregivers may access usual support services such as medical social worker consultations, allied health professional consultations, and support group participation. Data on the utilisation of these other services will also be collected.

### ENABLE-SG intervention

**Fig. 3:**
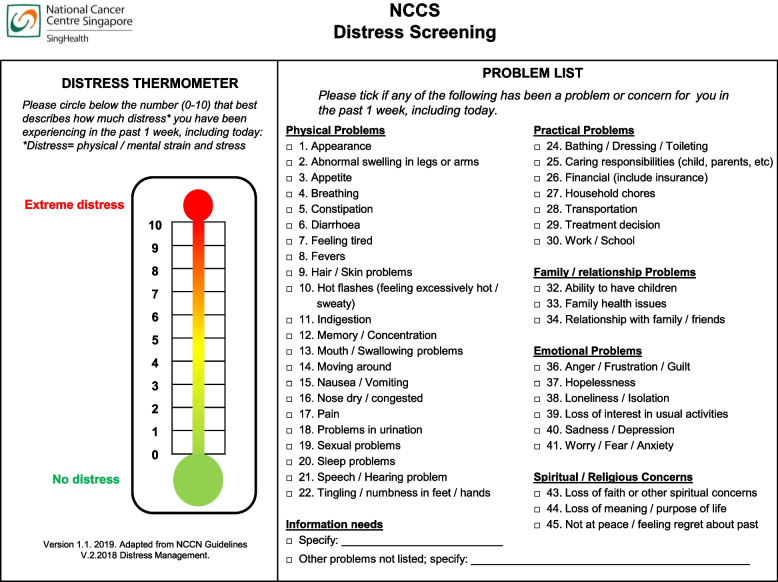
Distress Thermometer and Problem List culturally adapted from the National Comprehensive Cancer Network

ENABLE-SG closely follows the original ENABLE model with some modifications following cultural adaptation: (1) adding optional discussion topics, (2) assessing preferences for decision-making and tailoring sessions to individual family dynamics, and (3) allowing a flexible mode of delivery [32, 35]. Every patient and caregiver will receive six and four individual structured psychoeducational sessions, respectively, with a health coach. These sessions will be primarily delivered over the phone. Different health coaches will conduct sessions for each patient-caregiver dyad. The topics covered in the sessions are based on essential elements of early palliative care (Table 1) [18]. All sessions will begin with screening for distress using the Distress Thermometer and Problem List culturally adapted from the National Comprehensive Cancer Network (Fig. 3) [36]. From screening results, the health coach can flexibly change the order of the topics to address specific problems and discuss additional topics where relevant. The health coach will aim to conduct the sessions weekly. Most participants from the pilot study could complete the sessions within three months.

**Table 1:** Topics and elements of palliative care covered by ENABLE-SG sessions

Patients	Caregivers	Topics covered	Elements of palliative care
**Session 1** Maintaining positivity	**Session 1** Maintaining positivity	• Handling problems with a positive attitude • A problem-solving attitude • The seven steps of problem-solving	• Improving symptom relief and function • Psychosocial and spiritual care
**Session 2** Self-care	**Session 2** Self-care	• Healthy eating and nutrition • Exercise • Quitting smoking • Sexuality • Work and family	Improving symptom relief and function
**Session 3** Coping with stress	**Session 3** Coping with stress	• Coping with stress • Spirituality • Getting the support you need	Psychosocial and spiritual care
**Session 4** Managing symptoms	**Session 4** Managing symptoms	• Managing symptoms • Common symptoms in cancer • Common thoughts and feelings	Improving symptom relief and function
**Session 5** Talking about what matters most and making choices	-	• Talking with your family and healthcare providers • Core values: what matters most • Decision aids: making choices that are right for you	Enhancing communication, values-based treatment, and goals of care conversations
**Session 6** Life review	-	• Starting a conversation about your journey • Looking at today, looking at tomorrow • Creating a legacy	Psychosocial and spiritual care

### Intervention fidelity

For this study, ENABLE-SG will be delivered by a team of three to four health coaches from different healthcare backgrounds (nursing, social work, or allied health). Before deployment, all health coaches will be certified through the Health Coaching for Older Adults program offered by the Nanyang Technological University Singapore [37]. They will also receive ENABLE-SG-specific training, including independent readings, audio recordings demonstrating coaching techniques, study protocols and procedures, and role-play of six training cases. After deployment, the PI and a senior coach will review a random sample of 20% of recorded coaching sessions delivered by each health coach quarterly, using a standardized checklist to ensure fidelity to the coaching protocol. Less than 80% adherence to the study protocol will be grounds for coach remediation. Health coaches will further receive clinical supervision via weekly case discussions with a specialist palliative care nurse clinician, where the team may initiate a formal palliative care referral when appropriate.

### Data collection

At baseline, demographic information and clinical data will be collected from both patient and caregiver participants. Patient-reported outcome measures will be collected every 3 months until death or the end of the study. Caregiver-reported outcome measures will be collected every 3 months until the patient’s death or the end of study, whichever is earlier. All study outcomes are summarized in Table 2. Study team members will administer the questionnaires at in-person visits or over a phone call. A secure REDCap (Research Electronic Data Capture) database will be used to capture and store all data. All study team members performing collection, extraction, and entry of data will be blinded to the participants’ randomized group assignment. Data entry will be cross-checked by an independent study team member to ensure reliability and accuracy.

**Table 2:** Overview of study outcomes

Outcome	Participant	Instrument	Measure description	Assessment
***Specific aim 1***				
Health-related quality of life	Patient	FACIT-Pal	46-item measure; quality of life scale (4 domains – physical, emotional, social, and functional well-being) and a palliative care subscale. It has demonstrated internal consistency reliability and validity for persons with advanced cancer.	Baseline, every 3 months for 12 months
Mood	Patient	CES-D	20-item measure; symptom clustered in 4 domains – depressed affect, somatic complaints, positive affect, and interpersonal activity. The scale demonstrated adequate reliability and validity among community dwelling older adults in Singapore.	
Coping strategies	Patient	Brief-COPE	28-item measure; coping strategies in 14 subscales – self-distraction, active coping, denial, substance use, use of emotional support, use of instrumental support, behavioural disengagement, venting, positive reframing, planning, humour, acceptance, religion, and self-blame. This scale has been validated in the cancer population. It has also been used among family caregivers of individuals with dementia in Singapore before.	
Palliative care concerns	Patient	IPOS	Brief measure of palliative care problems, covering physical and psychological symptoms, social and spiritual issues, communication, information needs, and practical concerns. IPOS has been translated to Chinese and validated in Singapore.	
Health state	Patient	EQ-5D-5L	5-item descriptive system measuring 5 dimensions – mobility, self-care, usual activities, pain/ discomfort, anxiety/ depression; a visual analogue scale measuring overall health status. The tool has been validated in Singapore.	Baseline, every 3 months until death or end of study
Healthcare resource utilization	Patient	Electronic medical records	Dates of emergency department visits and hospital admissions, hospital bill size, date of first review by existing palliative care services, date of death, and place of death	Enrolment until death or end of study
***Specific aim 2***				
Health-related quality of life	Caregiver	SCQOLS	15-item measure; quality of life measure covering 5 domains – physical well-being, mental well-being, experience & meaning, impact on daily living, and financial well-being. It has been developed and validated in Singapore.	Baseline, every 3 months, for 12 months
Mood	Caregiver	CES-D	*Same as above*	
Coping strategies	Caregiver	Brief-COPE	*Same as above*	
Satisfaction with care	Caregiver	FAMCARE	10-item unidimensional scale measuring family satisfaction. It has been translated to Chinese and validated in Singapore	
Caregiving costs	Caregiver	Caregiving costs questionnaire	Employment status; productivity loss (hours missed from work, impairment while at work, and impairment in regular activities) due to caregiving	
		Electronic medical records	Dates of emergency department visits and hospital admissions, and hospital bill size	
***Specific aim 3***				
Acceptability	Patient Caregiver	Structured feedback survey	Acceptability, relevance, comfort level, overall satisfaction, and intention to recommend ENABLE-SG to others in similar situations are rated on a 5-point Likert scale	End of study participation
Adoption				
Appropriateness	Patient Caregiver Healthcare provider	Semi-structured interviews	Interview guide developed based on the Consolidated Framework for Implementation Research (CFIR)	≥ 6 months after baseline
Feasibility				
Fidelity	Health coach	Self-reporting checklist, field notes, recordings	Modifications to protocol, discussions from conducted coaching sessions	Throughout study period
Implementation cost	Health coach	Process maps, project records	Identify key intervention activities to elucidate cost of involved personnel and resources through a time-tracking system and an activity-based costing approach	
Penetration	Oncologist	Study records	Number and proportion of approached oncologists who agree for study to be conducted in their clinics	

*Abbreviations*: *Brief-COPE* Brief Coping Orientation to Problems Experienced Inventory, *CES-D* Center for Epidemiological Studies-Depression, *EQ-5D-5L* EuroQOL Group 5-Dimension Health-related Quality of Life Measurement, *FACIT-Pal* Functional Assessment of Chronic Illness Therapy – Palliative, *FAMCARE* Family Satisfaction with End-of-Life Care, *IPOS* Integrated Palliative Care Outcomes Scale, *SCQOLS* Singapore Caregiver Quality of Life Scale

#### Patient- and caregiver-reported outcomes

The primary outcome measure is patient health-related QoL measured using the Functional Assessment of Chronic Illness Therapy – Palliative (FACIT-Pal) at 6 months [38]. Secondary patient-reported outcomes include: (1) mood measured using the Centre for Epidemiological Studies-Depression (CES-D) [39, 40], (2) coping strategies measured using the Brief Coping Orientation to Problems Experienced (Brief-COPE) Inventory [41–45], (3) palliative care concerns measured using the Integrated Palliative Care Outcomes Scale (IPOS) [46], and (4) health status measured using the EuroQOL Group 5-Dimension Health-related Quality of Life Measurement (EQ-5D-5L) at 6 months [47–49]. Caregiver-reported outcomes include: (1) health-related QoL measured using the 15-item Singapore Caregiver Quality of Life Scale (SCQOLS-15) [50, 51], (2) mood measured using the CES-D, (3) coping strategies measured using the Brief-COPE, and (4) satisfaction with care measured using the Family Satisfaction with End-of-Life Care (FAMCARE) Scale [52].

#### Healthcare resource utilization, patient survival, and caregiving costs

The following data will be extracted from administrative and billing data in the electronic medical records for all participants: dates of emergency department visits and urgent hospital admissions, and gross hospital bills. For patients, additional data on the date of first review by existing palliative care services, date of death, and place of death will be extracted. Caregivers will further complete caregiving costs questionnaires during their 3-monthly follow-up on their employment status and productivity loss (hours missed from work, impairment while at work, and impairment in regular activities) due to unpaid caregiving.

#### Implementation outcomes

Proctor et al.’s taxonomy guided the selection of implementation outcomes [53]. The penetration of ENABLE-SG will be assessed by the number and proportion of approached oncologists who agreed to include their clinics for study screening. The acceptability, adoption, appropriateness, and feasibility of ENABLE-SG will be evaluated using a structured feedback survey completed by all participants at the end of study participation (or withdrawal) and semi-structured interviews with a purposive sample of patients, caregivers, and healthcare providers. Purposive sampling will ensure representativeness across ages, genders, cancer types, and the extent of ENABLE-SG completion. These semi-structured interviews will be conducted after the primary outcome measurement at 6 months post-enrolment. The interviewer will use an interview guide (Supplementary File 1) developed based on the Consolidated Framework for Implementation Research (CFIR) [54]. Each interview lasting approximately 30-60 min will be audio-recorded and transcribed verbatim. Recruitment for implementation outcome interviews will stop when data saturation is reached.

Fidelity will be assessed using self-reporting checklists, field notes on a structured template, and sample recordings of coaching sessions completed by each health coach. The weekly clinical supervision sessions will be recorded in field notes. Deviations from the ENABLE-SG protocol and reasons for modification will be deliberated and documented.

Implementation costs will be collected from the healthcare system’s perspective. Fixed set-up costs include health coach training (time, materials, space, supplies, trainer). To identify and value ongoing variable costs, we will develop process maps with health coaches to identify key activities (e.g., preparing, conducting, documenting, supervising, and auditing coaching sessions) to elucidate the personnel and resources involved using an activity-based costing approach [52]. We will record the pay scales of involved personnel and use a time-tracking system to record time spent in training and identified key activities. Time will be valued using hourly wages and fringe benefits at market rates for the services provided. The cost of materials and resources will be tracked and valued using project records or current market prices.

#### Sample size

We aim to detect an effect size of 0.4 standard deviation (SD) in the mean difference in FACIT-Pal total scores (primary outcome) between the two study groups 6 months after baseline. From our pilot data, 0.4 SD is equivalent to a mean difference of 10 points, reflecting a minimal clinically important difference estimated based on 5% of the maximal FACIT-Pal total score [55]. A sample size of 200 patient participants (both groups combined) will give 80% power at a 5% 2-sided type 1 error rate. Accounting for an attrition rate of 34%, typical of studies among patients with advanced cancer [19, 21, 29], we aim to recruit 300 patient participants (150 per group). Correspondingly, up to 300 caregiver participants will be recruited. The criteria to define sample size for implementation outcome interviews will be the point of data saturation where additional interviews do not yield new themes. From previous studies, this is estimated to be around 15 patients, 15 caregivers, and 15 healthcare providers.

### Data analysis

#### Analysis of effectiveness outcomes

Using an intention-to-treat approach, we will compare ENABLE-SG versus usual care at 6 months post-enrolment. Linear regression will be used to compare each patient- and caregiver-reported outcome between intervention and wait-list control groups, with baseline score as a covariate. The negative binomial regression will be used to compare the number of emergency department visits and number of days spent in hospital(s) during the first 6 months post-enrolment, with an offset term that equals 6 months or time from enrolment to death, whichever is earlier. Using a similar approach, we will compare early ENABLE-SG in the intervention group versus delayed ENABLE-SG in the wait-list control group for abovementioned outcomes at 12 months post-enrolment.

We will also assess between-group differences in outcomes from baseline across all timepoints from enrolment using a mixed-effects regression model for repeated measurements. Time since enrolment will be treated as a discrete variable that interacts with the trial groups [56]. Intervention effects will be estimated at each of the 3-monthly timepoints. The comparisons at 3- and 6-months post enrolment will indicate the effect of ENABLE-SG versus usual care; comparisons after 6 months will shed light on the effect of early versus delayed initiation of ENABLE-SG.

For patients, the date of the first specialist palliative care review will be used to compute the following indicators of the timing of palliative care initiation: (1) number of days from enrolment to first palliative care review; (2) number of days from first palliative care review to death. Survival and the timing of palliative care review initiation will be estimated and compared between trial groups using time-to-event analysis.

#### Analysis of implementation outcomes

Transcribed interviews will be analysed using a thematic analysis approach guided by the CFIR [54]. The study team will familiarize themselves with the data and context before performing deductive coding according to the CFIR framework. Passages will also be open-coded inductively to form code categories. Code categories will be systematically compared against one another to determine mutual exclusivity, clustering, or connectivity between categories. The dimensions and properties of concepts will then be developed. This process will repeat until patterns or themes emerge. Data will be coded independently by at least two coders to enhance the credibility of constructs derived from analyses. Regular team meetings will be conducted to resolve disagreements, document reflexive notes, and discuss interim findings.

Descriptive statistics will be used to describe responses from the structured feedback survey and data on fidelity. Counts and percentages will be used to summarize categorical variables. Mean and SD or median and interquartile range will be used to summarize normally distributed and non-normally distributed continuous variables, respectively. Additionally, for modified ENABLE-SG sessions, a narrative recount of what was modified, the underlying rationale, and the content will be presented. To assess penetration, the approach-to-participation rate will be tabulated at the oncologist level.

Implementation costs of ENABLE-SG will be captured prospectively using an activity-based costing approach. Non-sunk implementation costs will be combined with any potential cost offsets from improved health and used as the numerator in a cost-effectiveness analysis (CEA) from the health system perspective. The denominator of the CEA will focus on quality-adjusted life years (QALYs) gained, accounting for both differences in survival and health-related QoL resulting from ENABLE-SG. Survival differences will be estimated using time-to-event (death) analysis. QALY differences will be computed using EQ-5D-5L data at baseline, 3-, and 6-months post-enrolment. The resulting incremental cost-effectiveness ratio (ICER) will be compared to established thresholds for cost-effectiveness. One-way and probabilistic sensitivity analyses will also be constructed to gauge the variability of the results and the influence of select parameters and assumptions (e.g., survival duration, QoL benefits 6 months post-intervention).

### Ethics and dissemination

This study received ethical approval from the SingHealth Centralized Institutional Review Board (CIRB 2023/2462). Informed consent will be obtained from all participants. No formal monitoring committee was formed. The study team, led by the Principal Investigator, will be responsible for data monitoring to ensure data integrity, accuracy, and confidentiality. Secure databases will be used (REDCap, institutional database). All study data will only be accessible to authorized research personnel. All amendments reflecting study modifications will be submitted for institutional review board approval. The principal investigator declares no competing interests for the overall trial. We will present study results at national and international scientific meetings, publish them in peer-reviewed journals, and deposit the publication(s) in our institution’s open access repository within 12 months of publication. Authorship eligibility will abide by International Committee of Medical Journal Editors recommendations.

## Discussion

This study evaluates the effectiveness and implementation of a culturally adapted and piloted health coaching model (ENABLE-SG) as an early palliative care intervention for patients with advanced cancer and their caregivers. The ENABLE-SG model addresses existing gaps in current models of palliative care. First, early integration of palliative care in the patient's disease trajectory reverses the trend of palliative care being delivered late in the last weeks of life. Second, a proactive care approach shifts away from the current reactionary illness-stress paradigm, allowing patients to take charge of their health. By proactively equipping patients and caregivers with self-management skills, we anticipate that ENABLE-SG empowers them to cope better while living in the community, leading to better QoL and possibly lower acute healthcare utilization.

This study will generate evidence on the value of early palliative care provision on patient and caregiver outcomes and outline ways to support widespread ENABLE-SG implementation or its modification if needed. At the individual level, ENABLE-SG may facilitate a better coordinated and patient-centred approach to delivering supportive and palliative care to patients with advanced cancer and their caregivers. At the organizational level, insights into implementation processes will inform future upscaling and dissemination of this early palliative care model and bolster support for employing non-specialist, trained health coaches to deliver palliative care sustainably. Collectively, study findings may inform healthcare policy to promote early access to palliative care.

The study has several strengths. First, a wait-list control design represents a resource-efficient approach to investigating the impact of ENABLE-SG based on its contents and the timing of introduction. The 6-month wait-list duration is appropriate given that the median survival in the Singapore setting is 1.3 years in adults with stage 4 cancer and > 4 years in adults with stage 3 cancer. Second, the hybrid effectiveness-implementation design facilitates data collection from real-world settings to generate insights on factors influencing successful implementation in outpatient oncology clinics. Third, by primarily delivering the health coaching sessions over the phone, this study evaluates early palliative care provision through a telehealth delivery mode that is readily scalable in a lean healthcare system workforce.

## Conclusion

This hybrid type 1 effectiveness-implementation study evaluating the ENABLE-SG model aims to change how palliative care is provided – from a reactive illness approach to a proactive wellness paradigm. This proactive approach of empowering patients and their caregivers with self-management skills can reap long-term benefits for a broader spectrum of advanced illnesses beyond cancer. Moreover, insights into implementation processes can facilitate model expansion to benefit more patients and caregivers.

### Supplementary Information

**Additional file 1. **Interview guide for semi-structured interviews with patients, caregivers, and healthcare providers.

**Additional file 2. **Completed SPIRIT checklist comprising items to address in a clinical trial protocol and related documents.

## Data Availability

No datasets were generated or analysed during the current study.

## Abbreviations

Brief-COPE: Brief Coping Orientation to Problems Experienced
CES-D: Centre for Epidemiological Studies-Depression
CIRB: Centralized Institutional Review Board
CFIR: Consolidated Framework for Implementation Research
ENABLE: Educate, Nurture, Advise, Before Life Ends
ENABLE-SG: Educate, Nurture, Advise, Before Life Ends for Singapore
EQ-5D-5L: EuroQOL Group 5-Dimension Health-related Quality of Life Measurement
FACIT-Pal: Functional Assessment of Chronic Illness Therapy Palliative
FAMCARE: Family Satisfaction with End-of-Life Care
ICER: Incremental cost-effectiveness ratio
IPOS: Integrated Palliative Care Outcomes Scale
MCID: Minimal clinically important difference
NCCS: National Cancer Centre of Singapore
QALY: Quality-adjusted life year
QoL: Quality of life
REDCap: Research Electronic Data Capture
SCQOLS: Singapore Caregiver Quality of Life Scale
SD: Standard deviation
U.S.: United States
