# Supplementary material for: ENABLE-SG (Educate, Nurture, Advise, Before Life Ends for Singapore) as a proactive palliative care model: protocol for a hybrid type 1 effectiveness-implementation randomized wait-list controlled trial
Source: BMC Palliat Care. 2024 Jan 30;23:29. doi: 10.1186/s12904-024-01353-2 (PMC10826230; doi:10.1186/s12904-024-01353-2)
Supplement: Supplementary file 1 — Additional file 1. Interview guide for semi-structured interviews with patients, caregivers, and healthcare providers. [file 12904_2024_1353_MOESM1_ESM.docx]

**Interview Guide (Patient/ Caregiver)**

**Section 1: Overall impression of ENABLE-SG program**

1. What were your initial thoughts when joining our study?
   - How was the program introduced to you?
   - How did you make the decision to join this study/ program?
2. Overall, how did you find the program?
   - What was your favourite part?
   - What was one thing you dislike/ hope to change?
3. Did you attend similar programs before? E.g., coaching, education, counselling, etc.
   - How is ENABLE-SG similar and/or different?

**Section 2: Experience in ENABLE-SG program**

1. How did you prepare for the first session/ meeting with your health coach?
   - What are your expectations before the session? [*take note for Q5*]
2. How did the first session go? Could you bring me through what happened during the session?
   - Did you like the session format? Why?
     - How was the session conducted?
     - When was the session held?
     - How long was the session?
     - What language was it conducted in? Was it your preferred language?
   - Do you recall completing a screening tool [*check recall Distress Thermometer and Problem List*]?
     - Was it easy to complete?
     - How sufficient was the screening tool in assessing your needs?
     - How did your coach react to your responses?
   - What topic was discussed? How was the discussion?
     - How was the booklet used in the discussion?
     - Did you enjoy the activity/ exercise with the coach? How did it go?
     - Did you receive additional resources? What were these resources? Were they useful?
   - What are your first impressions of your coach? [*take note for Q7*]
     - Was it easy to talk to him/her?
     - How would you describe him/her? E.g., teacher, friend, healthcare professional.
   - What happen after the first session?
     - Did you use the booklet? Which parts did you read and why?
     - What thoughts or reflections do you have?
   - Just now you mentioned that your expectations of the session were [*responses to Q4*], were these expectations met? How were they met/ not met?
3. Now that we have talked about your experience in the first session, let’s now discuss about your experience in the subsequent sessions.
   - How did you prepare for the subsequent session/ meeting? [*note: compare with first session*]
   - What do you think about the format of sessions you had?
     - What do you think about the length of the sessions?
     - What do you think about the mode of the sessions?
     - What do you think about the total number of sessions?
   - How do you feel about completing the screening tool at every session?
     - What do you think about the frequency?
     - Do you think this screening is helpful/ necessary?
   - I would like you to think about the most memorable session you attended. Which session was it? Could you share with me what happened during the session?
     - Why was this session memorable? [*note: can be positive or negative*]
     - [*If negative*] What do you think we can do to improve the session?
     - [*If positive*] How can we make improve other sessions to be like this?
   - How did you (and your coach) use the booklet during the sessions?
     - How useful was the information in the booklet?
     - How can we make the booklet more appealing?
   - How did you (and your coach) engage with the activities during the sessions?
     - How did you enjoy the activities?
     - Were the activities useful?
     - How can we make the activities more engaging?
   - What are your impressions of your coach after all the sessions?
     - How did your impressions change from your first impressions [*responses to Q6*]?
     - How would you describe your relationship with your coach?
     - How easy was it to communicate with him/her?
     - Did you find your coach knowledgeable? How do you tell?
     - How did your coach help you?
   - What motivated you to finish the whole program [*or*] continue attending the sessions?
     - What were some of the challenges encountered?
     - How can we help you complete the sessions with more ease?
4. Do you discuss your experience in the program with your oncologists? [*or*] Did your oncologist ask you about your experience in the program?

- How did the discussion go? How did your oncologist respond?
- Would you have liked your oncologist to talk about the program with you? Why?
- What are your concerns with talking about your experience with your oncologist?

1. What was your takeaway from all sessions?
   - Are there any skills/knowledge you gained from the sessions that you are still practicing/using?
   - How did your perceptions of any topics change after the sessions?
2. Overall, how satisfied are you with the program?

**Section 3: Perceptions of program engagement and support**

1. What motivated you to finish the whole program [*or*] continue attending the sessions?
   - What were some of the challenges encountered?
   - How can we help you complete the sessions with more ease?
2. What do you feel about NCCS introducing this program to patients/ caregivers?

- Do you think this increase/ decrease the quality of care? In what way? Why?
- Do you feel that the program is well-supported? How do you tell? E.g., staff, resources, etc.

1. Would you recommend the program to other patients/ caregivers? Why?
2. How can we better engage patients/ caregivers like you to join this program?
   - What do you think could be some reasons why one might be interested to participate?
   - What strategies can be employed? E.g., by government, healthcare professionals, peers, etc.
   - What should we be mindful of?
3. Lastly, are there any other thoughts that you would like to share about the program or suggestions on how we can improve?

**Interview Guide (Healthcare providers)**

**Section 1: Experience with the ENABLE-SG program**

1. What do you know about the program?
   - How would you explain the program to your patient?
   - Who are involved? What is the intervention?
2. How would you describe your role in the implementation of this program?
3. Did you actively refer any of your patients/ caregivers to the study/ program?
   - How do you decide who to refer? What are your considerations?
   - What were some of the challenges encountered?
   - How can this process be improved?
4. Were you aware of any patient who was enrolled in this program?
   - Did you ask your patients about their experience with the program? [*or*] Did any of your patients actively share with you about their experience with the program?
     - How did the discussion go?
     - What did your patient share with you? How did you respond?
   - Can you share any stories about the patient experience that stood out to you?
   - Did you ask your patients about their experience with the program? [*or*] Did any of your patients actively share with you about their experience with the program?
     - How did the discussion go?
     - What did your patient share with you? How did you respond?
5. How did the implementation of this program affect your clinical practice?
   - Did any of your workflow process change?
   - Did the program disrupt your usual practice?

**Section 2: Perceptions of the ENABLE-SG program**

1. How do you feel about the program being introduced in your setting?
   - Were you excited, neutral, or un-enthused about implementing the program in your clinics? Why?
   - How much does this program fit within your organization and/or health system?
     - How can we optimize the program to be more compatible with our organization and/or health system?
   - Do you think this increase/ decrease the quality of care provided by your organization? Why?
2. Do you think the program is/ has been effective? How do you tell?
   - How would you describe the benefits of the program? E.g., to patients/ caregivers, healthcare staff, health system.
   - How essential is this program to meet the needs of patients/ caregivers in NCCS?
   - What kind of supporting evidence do you need to decide whether the program is effective?
   - How can we optimize the program to make it work more effectively?
3. Are you aware of similar programs implemented/ proposed in your setting?
   - Could you elaborate on the program you mentioned?
   - How is ENABLE-SG similar and/or different from the program you mentioned?
   - What are some possible advantages/ disadvantages of ENABLE-SG relative to others?
   - To what extent do you think your organization prioritize the implementation of ENABLE-SG?

**Section 3: Engagement with healthcare providers**

1. What do you think is the general level of receptivity in your department/ organization to implement the program?
   - How open is your department/ organization towards new initiatives/ programs?
2. How were you introduced to the program?
   - Who introduced the program?
   - Was the introduction engaging?
     - Was the information provided adequate? Any additional information you would have liked?
     - What were/ should have been emphasized?
     - How can we improve the introduction to promote program uptake among healthcare providers like you?
3. What level of endorsement or support have you seen or heard from your organizational leaders?
   - What kind of support or actions did these leaders demonstrate?
   - How did they influence your decision to uptake the program?
   - How did they influence your perception of the program?
4. Since the program is implemented, are you aware of any feedback sessions conducted among healthcare providers like yourself?
   - Could you share what happened during such a session?
     - What is the purpose of the session?
     - How was the session conducted? E.g., organizer, mode, duration, etc.
     - What information was shared?
     - What was discussed?
     - How did you participate in the discussion? What feedback did you provide?
     - How did the program lead(s) respond to your feedback?
     - Did you feel that your feedback was valued?
     - How responsive do you think the program lead(s) is/are?
   - How informed were you of the progress of the program implementation?
   - Were these feedback sessions useful?
5. Did you discuss this program with your colleagues?
   - What do you discuss about?
   - Did the discussion influence your perception of the program?
6. Do you think there was a champion for the program implementation? [*note: could be participant*]
   - What kinds of behaviors or actions did this individual/champion exhibit? [*or*] What kinds of behaviors or actions should a champion exhibit?
   - How were your interactions with this individual/champion?
   - How did this individual/champion influence your perception of the program?
   - How important/ useful is the presence of a champion for this program?

**Section 4: Promoting program sustainability**

1. Do you think the program is well-supported by resources? Why?
   - Tell me about the support or materials available. How accessible are they?
   - What do you think is the most resource-intensive component of the program?
   - How can we optimize the program to ensure program sustainability?
2. How can we promote the value of the program to our stakeholders? E.g., patients/ caregivers, department, organization, government, society, etc.
   - How can we motivate healthcare providers like you to adopt the program?
   - How can we engage our patients/ caregivers to participate in the program?
   - What role do you see yourself playing in program promotion?
     - What additional support will you need for this role?
3. Lastly, are there any other thoughts that you would like to share about the program or suggestions on how we can improve?
